# The effectiveness of mineralized plasmatic matrix in the closure of alveolar clefts with volumetric assessment

**DOI:** 10.1051/rmr/210004

**Published:** 2021-07-12

**Authors:** Padminii Ellapakurthi, Gotike Siva Prasad Reddy

**Affiliations:** Department of Oral and Maxillofacial Surgery, Panineeya Institute of Dental Sciences and Research Centre, Kamal Nagar, Dilshukhnagar −500060 Hyderabad Telangana State India

**Keywords:** Alveolar cleft defects, Oro-Nasal Fistula, SABG, PRP, PRF, volumetric assessment

## Abstract

*Objectives:* The purpose of this study is to assess the effectiveness of mineralized plasmatic matrix in the soft tissue closure of naso-alveolar fistula, to estimate the postoperative bone fill and volume of the graft placed in the alveolar cleft defect using cone-beam computed tomography (CBCT) at 3rd- month and 6th- month. *Material and methods:* 10 patients, in the age group of 15‑30 years were included in this study. They were diagnosed with unilateral cleft lip and alveolus defects with or without a cleft palate requiring late secondary alveolar bone grafting. Alveolar cleft defects were closed with mineralized plasmatic matrix (MPM), a combination of autogenous iliac bone graft and platelet rich plasma (PRP) and platelet rich fibrin (PRF). *Results:* The mean defect volume pre-operatively is 0.75 cm^3^ and at the end of 3rd-month postoperatively is 0.51 cm^3^ and at 6th-month postoperatively is 0.27 cm^3^. The average percentage of bone fill between preoperative (A) & 3th- month postoperatively (B) is 33.4% and between 3rd-month (B) and 6th-month post operatively (C) is 49.5%. *Conclusions:* Utilization of this new matrix (MPM), has shown to be effective in the closure of the cleft defect, oro-nasal fistula and also reduction in the volume of the residual cleft defect seen with sequential cone-beam computed tomography (CBCT) radiographs.

## 1 Introduction

An anterior osseous defect is found in 75% of all cleft lip and palate patients and is mostly associated with fistulas [1]. Bone grafting unifies the maxilla and is best done after the majority of facial growth is complete and during the period of secondary dentition. Normal facial and dental function of the alveolar defect is restored by a technique known as secondary alveolar bone grafting (SABG). This is a highly technique-sensitive procedure which needs proper reconstruction of the osseous deformity, failure of which may result in oronasal fistula, fluid reflux, speech pathology, anteroposterior and transverse deficiency of the maxilla, lack of bone support for the incisors and cuspids, dental crowding, and facial asymmetry. Several authors described SABG which was accepted as a means of uniting and stabilizing the segments of maxilla, prior to definitive orthodontic and restorative dental treatment [2]. This technique preferentially describes the usage of cancellous bone, which improves the craniofacial development and spontaneous eruption of adjacent teeth.

Autogenous bone grafting has been the choice for such procedures, as it affords the functions of osteoinduction, osteoconduction and osteogenesis. Anterior iliac crest has been the gold standard [3] for SABG besides the various options available like calvarial, rib, tibial and intra oral sites like chin, ramus and maxillary tuberosity. In some cleft patients it is difficult to augment the bone defect adequately. It is attributed to the complex morphology of the clefts and fistulas, timing of the graft placement, experience of the surgeon, making it more prone for oro-nasal fistulas, complete or partial resorption of the graft. To reduce these postoperative complications and to enhance the prognosis, we have incorporated the richness of both Platelet-rich plasma (PRP) and Platelet-rich fibrin (PRF) to the autogenous iliac graft. Growth factors PRP and PRF are under research for accelerating the speed of bone formation. PRP is concentrated white blood cells and platelets derived from whole blood centrifuged and separated from red blood cells. It enhances both bone formation and graft maturation. Similarly, PRF is a second-generation platelet derivative, which was found to stimulate human osteoblast growth and proliferation. PRF has a natural fibrin framework and can protect growth factors from proteolysis [4]. Growth factors can keep their activity for a relatively longer period and stimulate tissue regeneration effectively.

This special trio combination is called the mineralized plasmatic matrix (MPM), that is used in this study. This study has been supported with the 3-d volumetric analysis performed with cone-beam computed tomography (CBCT) along with clinical assessment.

## 2 Material and methods

10 patients attending Oral and Maxillofacial Surgery unit of Panineeya Mahavidyalaya Institute of Dental Sciences and Hospital, for the purpose of alveolar cleft defect closure were included in this study, from the time period of June 2016-to June 2019.

### 2.1 Inclusion criteria

- Patients in the age group of 15‑30 years.
- Patients with unilateral cleft lip and alveolus defects with or without a cleft palate requiring cleft alveolus closure.
- Patients/guardian consented for the procedure.

### 2.2 Exclusion criteria

- Patients with bilateral cleft lip & alveolus.
- Patients with associated syndromes.
- Patients with immunocompromised conditions.
- Patients having repeated bone grafting procedures.

### 2.3 Surgical procedure

**Fig. 1:**
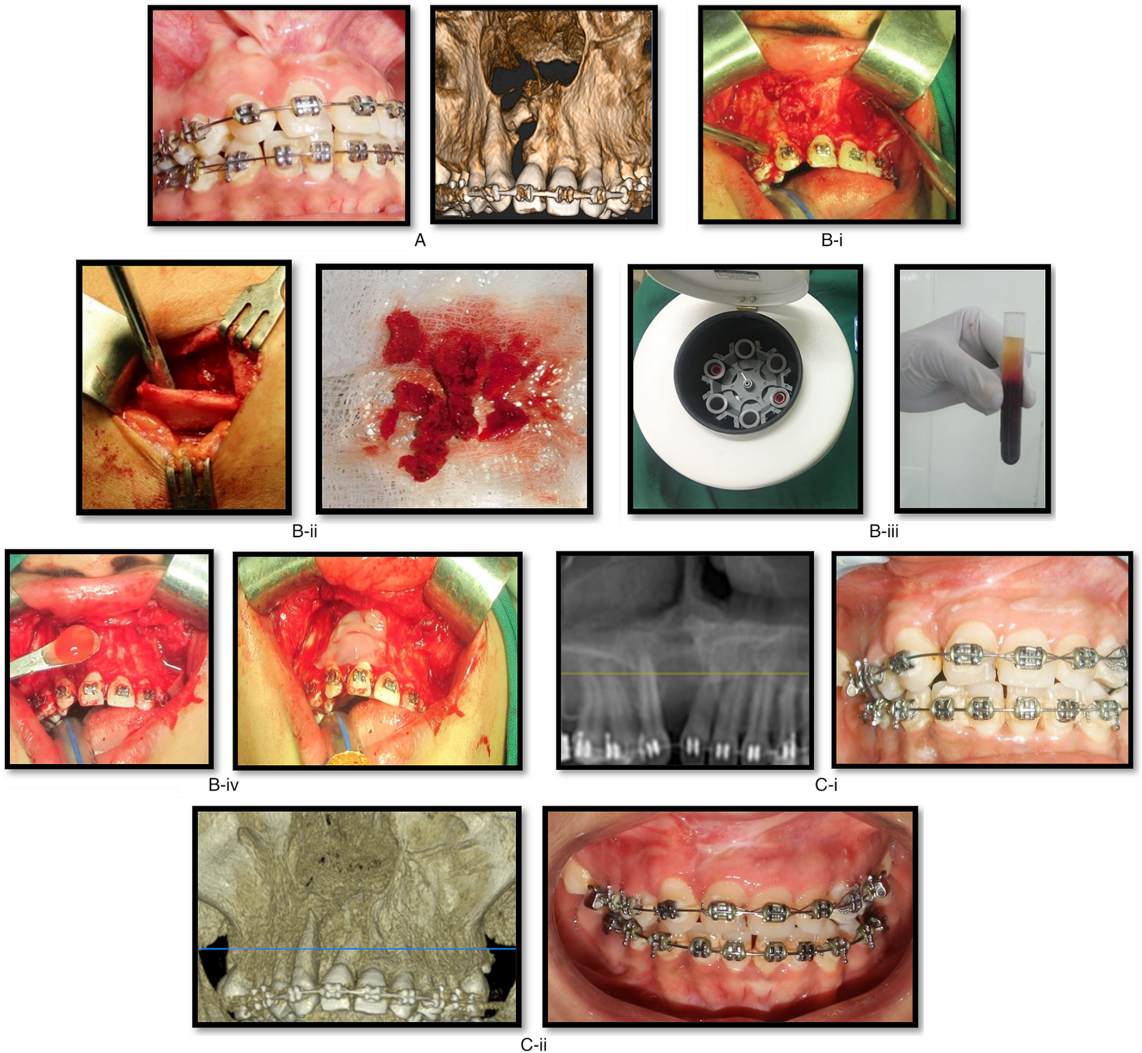
Case of cleft alveolus defect on the right side with oro-nasal fistula. (A) Preoperative images. (B) Intraoperative images. i) Exposure of cleft defect with fistula and preparation of recipient site; ii) Trap door approach for anterior iliac bone graft harvesting; iii) Preparation of PRP and PRF with autogenous blood in centrifuge. iv) Placement of bone graft with PRP and coverage with PRF membrane of the defect area. (C) Postoperative images. (i) 3rd −month postoperative images; (ii) 6th −month postoperative images.

Patients were explained about the surgical procedure and informed written consent was taken. Preoperative cleft defect was assessed by clinical examination & by a CBCT of the maxilla. All the cases were performed under general anesthesia with strict aseptic and sterile conditions. Standard oro-tracheal intubation is done and anesthesia was supplemented with local infiltration of 2% lignocaine hydrochloride with 1:80,000 adrenaline to achieve adequate hemostasis at the surgical site. The buccal, mucosal incision was placed along the cleft margins and around the teeth. The mucosa covering the cleft margins was detached along with delineation of nasal layer & sutured with 4–0 vicryl sutures. The graft bed was now ready. Incision placement for harvesting cancellous iliac bone graft, is 2 cm behind the anterior superior iliac crest to avoid iatrogenic damage to the lateral femoral cutaneous nerve. Care is taken to avoid excessive traction of the nerve, as it might result in meralgia paresthetica. Skin incision followed by muscle dissection; bone was accessed with a trap-door opening. The cancellous bone is harvested using a curette or gouge and stored in blood. The graft donor site is packed with gel-foam soaked in plain bupivicaine to minimize postoperative pain and the closure is done in layers with 3–0 vicryl and 3–0 prolene sutures. The cleft alveolus defect which is exposed, is filled with cancellous bone mixed with PRP, extended from the floor of the nasal cavity to the full height of the alveolar ridge. The graft is covered by the PRF membranes in the frontal aspect just underneath the vestibular flap acting as a protective layer [5] and a primary closure is done with 3–0 vicryl sutures and hemostasis is achieved (Fig. 1A–C).

### 2.4 Preparation of platelet-rich plasma (PRP) & platelet-rich fibrin (PRF)

Blood samples of the anesthetized patients were collected in 2ml vacutainers (2–4 per patient) without anticoagulant and immediately centrifuged at 3000 rpm for 10 minutes for PRF [6]. PRP is obtained from collecting the whole blood in anticoagulant containing citrate phosphate vacutainers. It undergoes an initial low and short time spin in the centrifuge which would separate red blood cells and plasma. After which a high and long-time spin is set so that platelets rich plasma is precipitated. At the time of grafting, it is mixed with 2% calcium chloride to trigger gelation and promote release of growth factors. Later this cancellous bone is mixed with PRP and grafted in to the recipient site.

**Fig. 2:**
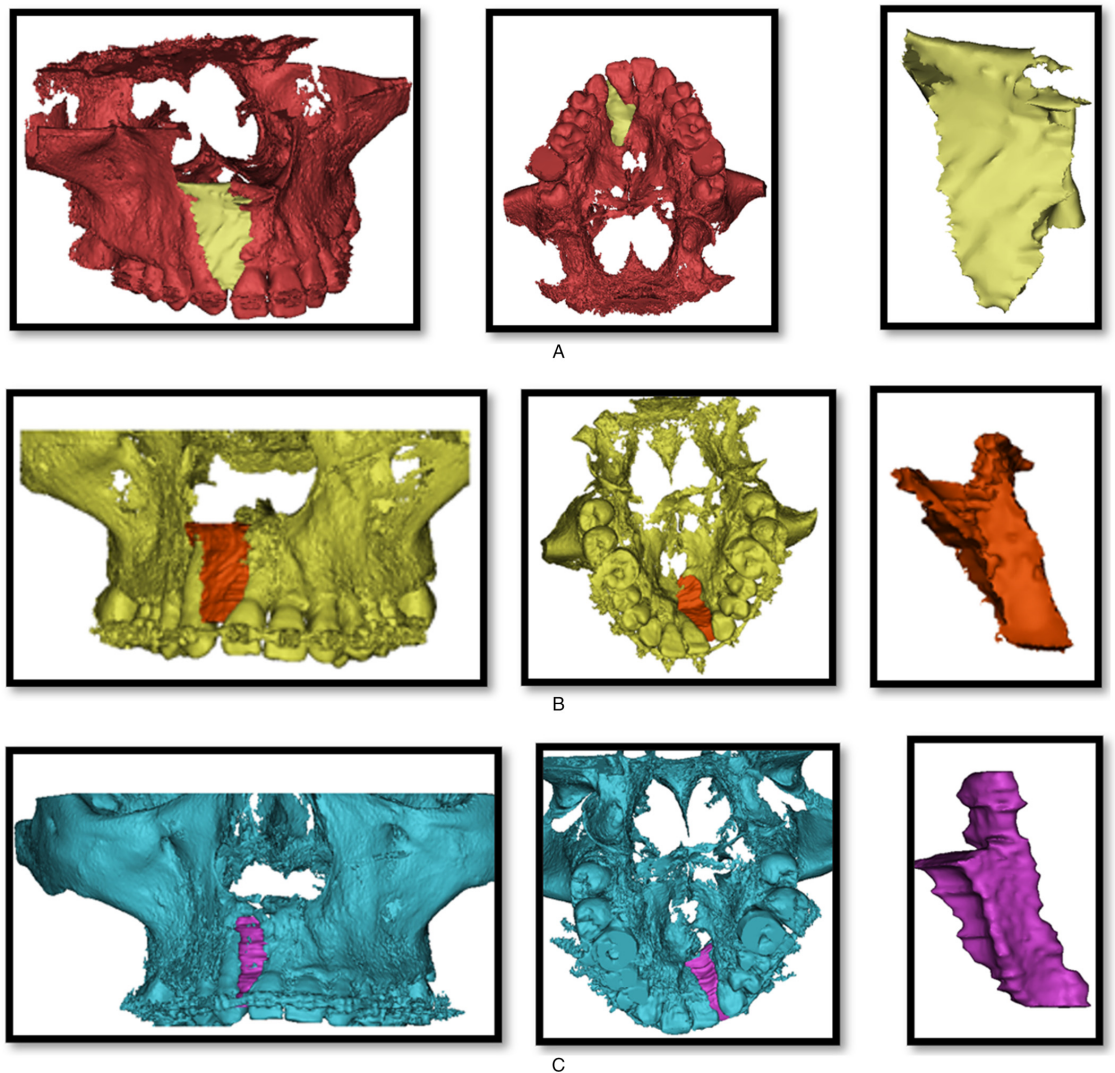
Volumetric analysis of the residual cleft defect of the same patient. (A) Preoperative images. (B) Intraoperative images. (C) Postoperative images.

In our study HDX WILL CBCT machine was used to achieve 0.1 mm thickness slices. The radiographic images were obtained in DICOM format. These images were further processed in the MIMICS MEDICAL 20.0 version software. The volumetric difference in the residual cleft defect before and after the closure with MPM was calculated (Fig. 2A‑C). The measurements were standardized in such a way, that the processed software images revealed the outline of the bone deficient area.

$$\text{The}\text{average}\text{Percentage}\text{of}\text{bone}\text{fill}\text{is}\text{calculated}\text{as}=\frac{\left(\text{Preoperative}\text{bone}\text{fill})-(3\text{rd}-\text{month}\text{bone}\text{fill}\right)}{\text{Preoperative}\text{bone}\text{fill}}\times 100$$

All the patients, were assessed with respect to closure of oro-nasal fistula, stability of alveolar arch and teeth adjacent to cleft area, infection, dehiscence, graft exposure, graft rejection, bone fill of the cleft area during the 3rd and 6th- month follow up period.

## 3 Results

Preoperative observations:

10 patients, 5 Male and 5 Female, have undergone late secondary alveolar bone grafting with iliac bone, PRP and PRF (MPM). Out of 10, 8 had palatal/ lingual alveolar fistulas,1 had labial alveolar fistula and 1 had at the junction of primary and secondary palates.

Intraoperative observations:

In one case, minor bleeding was encountered during harvesting of anterior iliac bone graft but hemostasis was successfully achieved using electro-cautery.

**Fig. 3:**
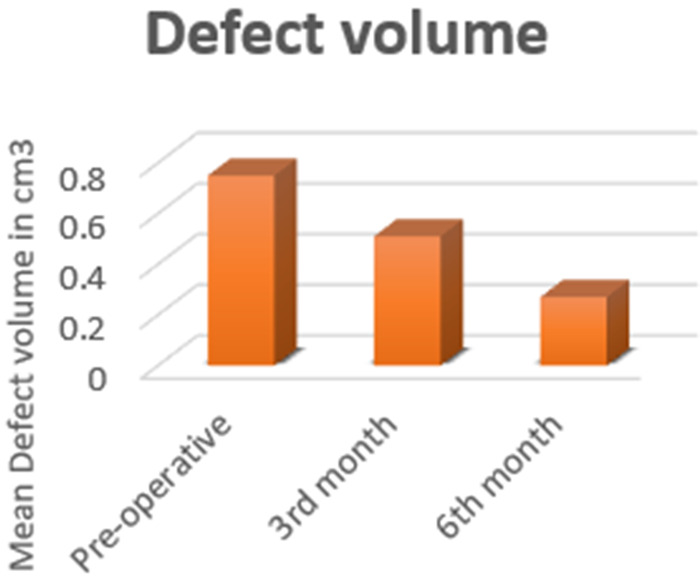
Alveolar cleft defect volume in cm^3^.

Postoperative observations:

In all the patients, closure of oro-nasal fistula was attained and stability of the alveolar arch and teeth adjacent to cleft area is observed. Infection was observed in 2 patients at the end of 3rd-month evaluation postoperatively, which resolved on use of antibiotics for 5 days and good oral hygiene maintenance. But subsequently, dehiscence and exposure of the graft, were also noted in the same patients, for which the exposed dead bone graft pieces were removed and thorough saline and betadine irrigation was done. Healing by secondary intention is noticed. No cases of graft rejection were observed. Bone fill at the end of 3rd and 6th −month has shown significant increase from initial assessment (Fig. 3).

The comparative analysis of the initial bone defect and the increase in the bone fill at 3rd and 6th-month post operatively is tabulated (Tab. 1). The one-way analysis of variance (ANOVA), establishes a statistically significant difference (*p* < 0.05) in residual cleft defect volume at three different time intervals (pre-operative group, 3rd-month group and 6th-month group) (Tab. 2). This table monitors the steady increase in the bone formation, optimizing the objective of enhancement of quantity and quality of bone fill with the trio matrix combination.

**Table 1:** Average percentage bone fill at the end of 3rd and 6th month.

Observation	Average % of Bone fill	Bootstrapped 95% CI
Between Pre- operative (A) & 3rd month (B)	33.4%	28.6‑38%
Between Pre-(A) operative & 6th month (C)	65.7%	58.4‑72.8%
Between 3rd month (B) & 6th month (C)	49.5%	41‑57.1%

**Table 2:** One-way ANOVA analysis for Cleft defect volume at different time intervals.

Descriptives														

Residual volume														

		N	Mean		Std. Deviation	Std. Error		95% Confidence Interval for Mean				Minimum		Maximum
							
								Lower Bound		Upper Bound				
Pre-operative		10	0.750		0.2273	0.0719		0.587		0.913		0.5		1.0
Third month		10	0.510		0.2025	0.0640		0.365		0.655		0.3		0.8
Sixth month		10	0.270		0.1567	0.0496		0.158		0.382		0.1		0.6
Total		30	0.510		0.2759	0.0504		0.407		0.613		0.1		1.0

**ANOVA**														

Residual volume														

				Sum of Squares			Df		Mean Square		F		Sig.	

Between Groups				1.152			2		0.576		14.741		0.000	
Within Groups				1.055			27		0.039					
Total				2.207			29							

## 4 Discussion

Restoration of the alveolar arch is a very crucial step in the management of cleft patients. Secondary alveolar bone grafting with anterior iliac crest is the gold standard method to restore such defects. PRP and PRF have been studied individually with the autogenous bone graft but a combination of both, has been explored less. According to a research on ‘Alveolar bone graft with Platelet-Rich Plasma in cleft alveolus’, PRP has enhanced bone formation and density in alveolar clefts when admixed with autologous bone chips harvested from the iliac crest [7]. PRP causes early vascularization, bringing in circulating macrophages and neutrophils and create a more oxygen-rich environment. It acts as a fibrin glue and the adhesiveness of this glue promotes better flap adaptation and hemostasis. Being acidic in nature, causes low infection rate and least postoperative complications. Some authors have [8] established positive effects of PRF on cell attachment, proliferation, phosphorylated Akt, heat shock protein 47 (HSP47) and lysyl oxidase (LOX) expression on human osteoblasts and the bone regeneration process. Similar results have been procured in this study (Tab. 3), in matters of the progressive increase and higher quality turnover of bone fill seen with the coalescence of PRP and PRF with the gold standard iliac graft. The late secondary bone grafting age groups, are more prone to complications like graft resorption, graft rejection etc. PRP enhances bone remodelling in the early phase [9], however, PRP alone seems to be insufficient as a counter measure against bone resorption following secondary bone graft in the long term. Eriko et al. [10] proved the reduction of bone resorption by the application of platelet-rich plasma (PRP) in bone grafting of the alveolar cleft. They also emphasized that PRP was effective in preserving the width and height of the graft than autogenous cancellous bone graft alone. MPM is totally autologous and thus devoid of risks of infection and rejection when used along with bone grafting [4]. It is a cost-effective source of growth factors and is an easy and effective alternative to be considered for the treatment of alveolar clefts.

**Table 3:** Patient data treated with mineralized plasmatic matrix (anterior iliac bone grafting with PRP and PRF.

Patient	Age (yrs.)	Sex	Primary Diagnosis	Complications
1	22	F	Unilateral right cleft lip, alveolus and palate	Not significant
2	28	M	Unilateral left cleft lip and alveolus	Not significant
3	22	M	Unilateral left cleft lip and alveolus	Infection, dehiscence, partial graft exposure
4	23	F	Unilateral left cleft lip and alveolus	Infection, dehiscence, partial graft exposure
5	22	M	Unilateral left cleft lip, alveolus and palate	Not significant
6	21	F	Unilateral left cleft lip, alveolus and palate	Not significant
7	26	M	Unilateral left cleft lip, alveolus and palate	Not significant
8	17	F	Unilateral left cleft lip, alveolus and palate	Not significant
9	25	M	Unilateral Right cleft lip, alveolus and palate	Not significant
10	18	F	Unilateral Right cleft lip, alveolus and palate	Not significant

Many researchers have confirmed that autogenous iliac bone graft is the gold standard for closure of clefts, such studies were supported with two-dimensional radiography. This still doesn't answer the question of, how much bone graft is needed to procure? The quantity determination for the surgeon, mostly was on the table and by experience. This would lead to increase in complications like, donor site morbidities, postoperative recovery of the patient, paraesthesia and much more. This issue is addressed in this study by utilizing the 3-dimensional volumetric assessment. A presurgical volume measure, was useful for the surgeon to determine amount of graft material needed and also to estimate the percentage of bone fill postoperatively. Snehlata et al. [11] conformed the use of CBCT radiographic outcome of secondary alveolar bone grafting in individuals with non-syndromic unilateral or bilateral cleft lip and palate using cone beam computed tomography with volumetric analysis and concluded that CBCT is a reproducible and shows adequate bone fill post operatively. Numerous studies in literature have produced data regarding the preoperative alveolar cleft defect size to range approximately from 1.3 to 2.1 cm^3^. When compared to our study, the mean defect volume preoperatively is 0.75 cm^3^ and at the end of 3rd-month post operatively is 0.51 cm^3^ and at 6th-month postoperatively is 0.27 cm^3^. This establishes a more affirmative response of PRP and PRF when utilized along with autogenous iliac bone graft. The percentage of bone fill in this study is 33.4% at the end of 3rd month with the range of 28.6‑38% confidence interval and at the end of 6th month is 65.7% with the range of 58.4‑72.8% confidence interval when compared with preoperative cleft defect volume. This study has put into reconnoitre, the use of PRP and PRF along with the autogenous iliac bone graft. PRP is proved to enhance the osteogenesis of alveolar bone grafting in cleft lip and palate patients [12]. The concept of incorporation of activated growth factors, locally (PRP and PRF), has an influence on the bone quality and quantity that has been grafted into the defect, when compared to grafting it alone. The acessibility and availability of these growth factors, makes it more easier for the surgeons to readily implement such modifications in the regular practice.

## 5 Conclusion

This study can be used as a template for new modifications to be included in the established regimen, encouraging us to provide improved patient care. Future comparative studies with larger sample sizes, and other parameters like unilateral and bilateral cleft alveolar defects, early secondary alveolar bone grafting, impacted tooth in cleft area, recurrent failure of closure of fistulas must be considered to give a definitive conclusion, for use of this procedure as a routine protocol.

## Disclosure statements

The authors report no conflicts of interest related to this study.
